# Growth Spectrum Complexity Dictates Aromatic Intensity in Coriander (*Coriandrum sativum* L.)

**DOI:** 10.3389/fpls.2020.00462

**Published:** 2020-05-15

**Authors:** Lorna McAusland, Mui-Ting Lim, David E. Morris, Hayley L. Smith-Herman, Umar Mohammed, Barrie R. Hayes-Gill, John A. Crowe, Ian D. Fisk, Erik H. Murchie

**Affiliations:** ^1^Division of Plant and Crop Sciences, School of Biosciences, University of Nottingham, Nottingham, United Kingdom; ^2^Division of Food Sciences, School of Biosciences, University of Nottingham, Nottingham, United Kingdom; ^3^Bioengineering Research Group, Faculty of Engineering, University of Nottingham, Nottingham, United Kingdom; ^4^Optics and Photonics Group, Faculty of Engineering, University of Nottingham, Nottingham, United Kingdom

**Keywords:** coriander, vertical farming, aroma, spectrum, light-emitting diodes, photosynthesis

## Abstract

Advancements in availability and specificity of light-emitting diodes (LEDs) have facilitated trait modification of high-value edible herbs and vegetables through the fine manipulation of spectra. Coriander (*Coriandrum sativum* L.) is a culinary herb, known for its fresh, citrusy aroma, and high economic value. Studies into the impact of light intensity and spectrum on *C. sativum* physiology, morphology, and aroma are limited. Using a nasal impact frequency panel, a selection of key compounds associated with the characteristic aroma of coriander was identified. Significant differences (*P* < 0.05) were observed in the concentration of these aromatics between plants grown in a controlled environment chamber under the same photosynthetic photon flux density (PPFD) but custom spectra: red (100%), blue (100%), red + blue (RB, 50% equal contribution), or red + green + blue (RGB, 35.8% red: 26.4% green: 37.8% blue) wavelengths. In general, the concentration of aromatics increased with increasing numbers of wavelengths emitted alongside selective changes, e.g., the greatest increase in coriander-defining E-(2)-decenal occurred under the RGB spectrum. This change in aroma profile was accompanied by significant differences (*P* < 0.05) in light saturated photosynthetic CO_2_ assimilation, water-use efficiency (W_i_), and morphology. While plants grown under red wavelengths achieved the greatest leaf area, RB spectrum plants were shortest and had the highest leaf:shoot ratio. Therefore, this work evidences a trade-off between sellable commercial morphologies with a weaker, less desirable aroma or a less desirable morphology with more intense coriander-like aromas. When supplemental trichromatic LEDs were used in a commercial glasshouse, the majority of compounds, with the exception of linalool, also increased showing that even as a supplement additional wavelength can modify the aromatic profile increasing its complexity. Lower levels of linalool suggest these plants may be more susceptible to biotic stress such as herbivory. Finally, the concentration of coriander-defining aromatics E-(2)-decenal and E-(2)-hexenal was significantly higher in supermarket pre-packaged coriander leaves implying that concentrations of aromatics increase after excision. In summary, spectra can be used to co-manipulate aroma profile and plant form with increasing spectral complexity leading to greater aromatic complexity and intensity. We suggest that increasing spectral complexity progressively stimulates signaling pathways giving rise to valuable economic traits.

## Introduction

Light-emitting diodes (LEDs) were introduced in 1962, but have only recently made great progress in revolutionizing the growth of horticultural crops (Davis and Burns, 2016).

LEDs enable growers to maintain profitability in a highly competitive market by reducing the space needed for each plant as well as consuming less energy and having longer lifespans than their traditional counterparts (Massa et al., 2008). While these benefits represent the logistical advantage of LEDs over conventional grow lights (e.g., high-pressure sodium), perhaps their most interesting application lies in the ability of the grower to manipulate crop characteristics through the modification of spectra. Controlling the contribution of key wavelengths allows the grower to manipulate not only the quantity, but also the quality of their crop, for example; the aesthetic appearance (color, flowering time), disease susceptibility, taste, smell, and nutritional content (Dudareva et al., 2006; Samuolienë et al., 2012; Davis and Burns, 2016; Alrifai et al., 2019; Pennisi et al., 2019).

Plants convert or transduce the energy of absorbed photons into biochemical constituents using photosynthetic pigments and photoreceptors (Kong and Okajima, 2016). Utilizing photons from the photosynthetically active radiation (PAR) region of the electromagnetic spectrum (wavelengths between 400 and 700 nm), photosynthetic pigments such as chlorophylls and associated accessory pigments (e.g., carotenoids) generate high energy electrons from water that are ultimately used to fix CO_2_ (Demarsy et al., 2018). While greater PPFDs and therefore higher rates of CO_2_ fixation, leading to greater acquisition of biomass, excessive light can result in damage to components of the photosynthetic system (Takahashi and Badger, 2011). In contrast, photoreceptors determine plant structure and morphology and can operate within and outside of the PPFD region—for example, phototropins perceive both blue (400–500 nm) and UV-A (315–400 nm) wavelengths—mediating processes such as phototropism (Briggs and Christie, 2002; Fraser et al., 2016). Three major photoreceptors regulate the development and growth of the plant dependent on the perception of the environment; the phytochromes, cryptochromes, and phototropins (Larner et al., 2008). These receptors respond to red:far-red ratio and blue wavelengths, respectively. No single receptor has been established for green although the cryptochromes absorb wavelengths in the green region in addition to blue (Smith et al., 2017). Variation in the ratios of these wavelengths affects diverse biological processes, for example, it is well known that photoreceptors have a major role in the determination of desirable morphological features in crops such as leaf area, leaf thickness, and internode length which help construct a canopy for efficient light interception (Crepy and Casal, 2015; Fraser et al., 2016; Smith, 2017). However, different wavelengths are also known to regulate chemical biosynthesis pathways; for example, stimulating the production and subsequent accumulation of carotene (Samuolienë et al., 2012, 2016) and phenolics (Lee et al., 2016) in some species. However, there is less information on the impact of spectral quality on the complex traits of flavor and aroma in horticultural crops such as herbs (despite their high value) with research tending to focus more on lettuce (Zhang et al., 2019), tomato, and bedding plants (Colquhoun et al., 2013).

Coriander is prized globally as a culinary ingredient and for its medicinal properties (Diederichsen, 1996; Sahib et al., 2013). While the seeds are an essential ingredient for products such as curry paste, the leaves and stems are also consumed in sauces and chutneys and added fresh to salads and garnishes due to their attractive green color and fresh citrus aroma and taste. Marketed in small pots or fresh leaves in packaging, coriander is the biggest selling fresh herb in the United Kingdom, with over 1500 hectares cultivated and over 30 million packets sold in 2014 (57 min^–1^, Fraser et al., 2015)—generating an estimated gross market value of £23.7 million. With a large proportion of this crop grown under controlled glasshouse conditions, it lends itself to the application of supplemental LED lighting for improved quality in the light limited northern hemisphere.

The aroma of coriander is critical to its product value. Previous work has shown that corianders distinctive aroma is determined by a combination of volatiles released from its essential oils; most notably the aliphatic aldehydes (Fan and Sokorai, 2002). In the literature, a selection of compounds has also been identified and described, including (but not limited to) 7-dodecenal, dodecanal, decanal (Macleod and Islam, 1976; Curutchet et al., 2014) and linalool. From the literature, E-2-decenal has been reported as the most abundant compound found in coriander leaves (Eyres et al., 2005). These compounds have different odour perception thresholds; abundance does not always imply olfactory impact. The released volatiles from crushed coriander leaves can be characterized based on detection of odour-active compounds with GCO and a human sniffer (Eyres et al., 2005). From the literature, coriander aromatics from many countries have been determined—from Africa (Buthelezi et al., 2016), Fiji (Eyres et al., 2005), and India (Shahwar et al., 2012).

While some publications accurately describe the growing conditions of the sampled coriander (Buthelezi et al., 2016), some do not (Macleod and Islam, 1976; Eyres et al., 2005; Shahwar et al., 2012). It is important to recognize that environmental conditions (e.g., light, temperature, watering regime), origin, and material age all contribute to the final aroma profile of the plant (Dudareva et al., 2006).

Here we investigate the role of growth wavelength on the aroma and morphology of coriander shoots grown in the United Kingdom. The fresh coriander product delivered to the supermarket in packets or pots, consists largely of a bushy, leafy canopy with multiple thin stems, both of which are used in food preparation. The coriander ideotype would be a short plant with high fresh biomass and high leaf:stem ratio, all of which are modulated by growth spectrum. For example, a higher proportion of blue can lead to smaller bushier plants but may increase the thickness of the stems making them less palatable. We hypothesize that growth spectrum will also influence the composition of flavor and aroma compounds and this may be advantageous or detrimental factor for the grower. For example, it may decrease the desirable coriander aroma or flavor. Conversely, using specific-spectra LEDs to grow coriander in the United Kingdom may represent an opportunity to improve quality and marketability if the trade-offs between morphology and aroma/flavor are minimal.

To test this hypothesis, we grew coriander under controlled growth conditions, manipulated the spectra, and determined the content of key aromatic compounds, which had the greatest impact on aroma via gas chromatography (GC) profiles. We also analyzed whole plant physiological traits of the specific-spectra grown plant, such as morphology, biomass, absorbance, and photosynthetic capacity to determine how specific growth spectra would impact the desirable, morphological characteristics of coriander. We show that growth spectrum has a significant impact on the content of desirable aromatic compounds in above-ground material and that there are both potential trade-offs and opportunities for using LEDs to manipulate morphology and quality in this species of herb.

## Materials and Methods

### Plant Material

#### Plant Growth Conditions and LED Treatments

**FIGURE 1:**
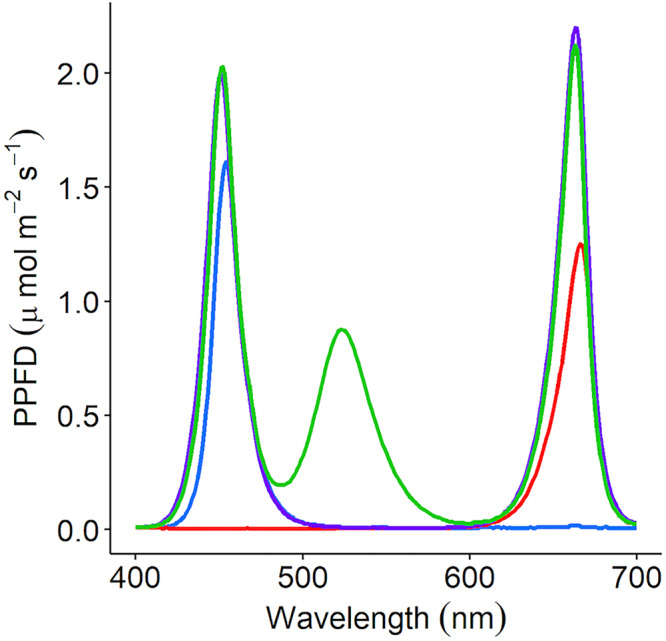
*C. sativum* was grown under four separate irradiance spectrums using LED lamps; red (peak: 666nm), blue (peak: 454 nm), RB [red (663 nm) + blue (451 nm)], and RGB [red (663 nm) + green (523 nm) + blue (451 nm)]. Each lamp was adjusted to supply 150 μmol m^–2^ s^–1^ photosynthetic photon flux density (PPFD). The supplemental LED lights used in the commercial glasshouse output the red + green + blue (35.8% red: 26.4% green: 37.8% blue—RGB) spectrum.

*Coriandrum sativum* (“Green Aroma”) was grown in a controlled environment chamber at 23/19°C light/dark and 60% relative humidity (RH). The photoperiod was 16/8 h light/dark with a PPFD of 150 μmol m^–2^ s^–1^; 20–30 seeds were sown per 250 mL pot with each pot containing compost (Levington’s F2S, Everris, Ipswich, United Kingdom). Pots were placed under either monochromatic (red or blue), dichromatic (RB), or trichromatic (35.8% red: 26.4% green: 37.8% blue) wavelengths (Figure 1) and grown for 21 days under well-watered conditions. The LED lamps were designed in-house by electrical engineers at the University of Nottingham. LED components were supplied by Prolight Opto Technology Corporation (Taipei, Taiwan). These were Pk2NThsi series: red (623 nm), green (525 nm), blue (465 nm), and cherry red (far-red) (730 nm).

For GC analysis, samples were pooled per pot; all above-ground tissue was harvested by grinding into a fine powder in liquid nitrogen and stored in −80°C until analysis. Analysis was performed on 3 g of the macerated tissue from four independent pot replicates. Concurrently, a second batch of four pots was sown for physiological, morphological, and destructive harvest measurements. Germination success and height were monitored. At day 21, an infra-red gas analyzer (GFS-3000, Heinz-Walz, Germany) was used to measure the response of the leaves to step-changes in light intensity between 0 and 2000 μmol m^–2^ s^–1^ PPFD (red with 10% blue provided by LEDs in the GFS-3000). These response curves were adjusted for leaf absorbance (400–700 nm) as determined using an integrating sphere (Li-1800, Licor Biosciences, Lincoln, NE, United States) and spectroradiometer (ASD Handheld, Malvern Panalytical, Cambridge, United Kingdom). The light response curves were fitted in R (RStudio Team, 2015) following the method of Marshall and Biscoe (1980) and Heberling and Fridley (2013). The following light response curve parameters were estimated from fitted non-rectangular hyperbola; A_sat_, ETR (electron transport rate as calculated using the equations of Genty et al., 1989), apparent quantum yield (the linear relationship between number of photos absorbed and μmol CO_2_ assimilated), R_d_ (R_d_, the daytime dark respiration rate), curve convexity (the efficiency of *A* under intermediate light levels), and the LCPT [light compensation point (LCPT)—the light intensity where rates of *A* and R_d_ are equal]. All plants were harvested and analyzed for fresh and dry weight and leaf to stem ratio.

#### Determining the Impact of Supplemental LED Lighting in a Commercial Glasshouse

To determine the impact of supplemental LED lighting on aroma in a commercial setting, *C. sativum* was grown in an automated 1.2 hectare commercially set controlled glasshouse in West Sussex (Langmead Group plc, Chichester, United Kingdom) is a commercial setup producing pot coriander for UK supermarkets. The photoperiod was a 16/8 h light/dark cycle with temperature at 23/19°C. *C. sativum* seeds were sown in 250 mL pots containing a company compost mix and pre-germinated in the 550 m^2^ dark germination room for 2 weeks. Once seedlings reached 2 inches tall, the plants were placed either under supplemental LED light at emitting 150 μmol m^–2^ s^–1^ PPFD trichromatic spectrum (see Figure 1 for spectrum) positioned approximately 150 cm above the plants or without supplemental lighting. They were grown under well-watered condition. At 35 days old, samples were taken for GC analysis. The material from each pot was pooled for sampling and three separate pots were sampled for the aroma study.

#### Supermarket Coriander

To determine differences between fresh (potted) coriander and pre-excised leaf material (packed or bagged), three independent samples were purchased from a local supermarket (Tesco, Long Eaton, United Kingdom). It was assumed that each pot contained between 20 and 26 plants (13 seeds per pot) and that each packaged bunch of coriander was harvested from a single pot when processed. Three pots of coriander were purchased from a supermarket the night before the extraction and well-watered. It was ensured that the use-by date for the plants was < 5 days from the time of purchase. All above ground tissue was harvested for GC-O analysis to determine key aromatics that characterized the scent of coriander.

### Gas Chromatography Mass Spectroscopy (GC-MS)

#### GC-MS Sample Preparation

0.5 g frozen ground material and 2.5 g of saturated calcium chloride solution (extraction ratio 1: 5 w/w) was added into 20 mL amber SPME vial. Internal standard (3-heptanone) was also added for relative quantification purpose. Vials were sealed with a septum cap ready for GC-MS analysis.

#### GC-MS Analysis

Sample volatiles were analyzed on a Thermo Scientific GC Mass Spectrometer (TRACE 1300 GC, USA, and ISQ series MS).

Headspace volatiles were extracted for 20 min at 50°C, using 50/30 μm DVB/CAR/PDMS SPME Fiber (Supelco, Sigma–Aldrich, United Kingdom), following desorption for 0.2 min at 250°C in splitless mode. ZB-Wax capillary column (length 30 m, inner diameter 0.25 mm, and film thickness 1 μm) (Phenomenex Inc., Macclesfield, United Kingdom) was used with temperature programmed starting at 40°C for 2 min, 8°C/min to 240°C, and held for 5 min.

Helium was used as carrier gas at a constant pressure of 18 PSI and full scan mode scanned from *m/z* 35–300. Identification of volatile compounds by matching retention time of pure chemical compounds and comparing its mass spectrum against reference libraries (NIST/EPA/NIH Mass Spectral Library, version 2.0, Faircom Corporation, United States).

To determine the impact of the odour of each aromatic compound relative to its concentration the OAV was calculated (Eq. 1) as:

(1) $$O\,A\,V=\frac{c\,o\,n\,c\,e\,n\,t\,r\,a\,t\,i\,o\,n\,\left(p\,p\,b\right)}{o\,d\,o\,u\,r\,t\,h\,r\,e\,s\,h\,o\,l\,d}$$

The odour thresholds for each compound were sourced from the literature (Leffingwell and Associates, 1985).

#### GC-Olfactometry Procedure

**FIGURE 2:**
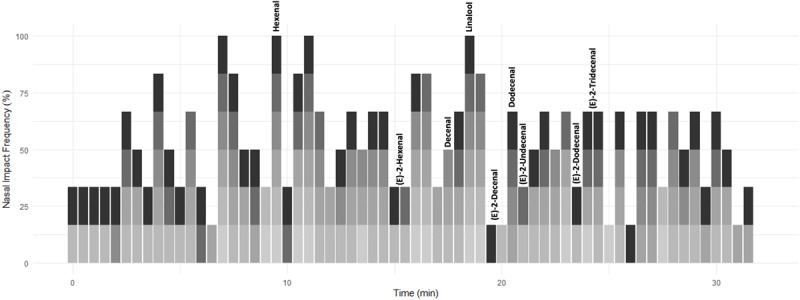
A time series depicting the time aromatic compounds were detected in commercially sold *C. sativum* by six panelists (denoted in shades of gray). The scent was assigned a score of 0 (no aroma detected) to 1 (aroma detected) over a period of 32 min and then converted to%. This annotated time series was compared to the GC-MS chromatogram of supermarket-purchased *C. sativum*. Aromatic compounds of interest are annotated.

To assess the impact of each of these compounds and to select the most important components for subsequent analysis, six judges sniffed samples from combined above-ground material from four pots (∼13 plants per pot) of *C. sativum* on a GC machine (TRACE 1300 GC, USA, and ISQ series MS) equipped with customized olfactometry detector outlet. The duration of each sniffing session was 32 min with panelists asked to note down when an odour was detected. The six individual aromagrams for the plant material from panelist treatment were added up to one aromagram (Figure 2). The NIF was calculated as the percentage of panelists detecting a scent at a particular time. In this way, peak height is not related to relative compound concentration but to frequency of compound detection. Hence, the compounds detected by the highest number of panelists represent the compounds that exert the greatest influence or impact to the overall odour of the coriander sample. The compounds identified from the NIF tests, and associated literature, were used to determine which compounds to be analyzed and quantified in subsequent chromatograms.

Descriptors and odour thresholds for all aromatic compounds determined from Flavornet (Acree and Teranishi, 1993; Acree and Heinrich, 2004; The Good Scents Company [tgsc], 2018; Brown, 2020), and Leffingwell and Associates (Leffingwell and Associates, 1985).

### Statistics and Experimental Design

Statistical analyses were conducted in R^1^. A Shapiro–Wilk test was used to test for normality and a Levene’s test of homogeneity was used to determine if samples had equal variance. Single factor differences were analyzed using a one-way ANOVA with a Tukey–Kramer honest significant difference (HSD) test where more than one group existed. The light response curves and derived parameters were determined using R according to the equations of Marshall and Biscoe (1980).

## Results

A NIF aroma gram was determined on potted supermarket coriander (Tesco Ltd., Long Eaton, United Kingdom) samples using a panel of six sniffers and GC-O (Figure 2 and Supplementary Figure 1). The key aromatics of coriander were selected based on NIF identification and the literature (Table 1) along with their corresponding odour thresholds. The odour threshold is defined as the lowest concentration of a compound that can be detected by the human nose (Van Gemert, 2003). If two compounds have the same concentration in the sample, the compound with the smaller odour threshold will potentially be identified over the compound with the larger threshold.

**TABLE 1:** Key aromatic compounds associated with the aroma of coriander, the odour threshold at which they are detected (ppb), and the generally accepted descriptor—these compounds were then taken forward for each analysis to determine differences in aroma between *C. sativum* grown under varying environmental conditions.

**ID**	**Aromatic compound**	**Odour threshold (ppb)**	**Descriptors**
1	Hexanal	4.5	Grass, tallow, fat
2	(Z)-3-Hexenal	0.25	Green, fatty, grassy, weedy, fruity, apple
3	(E)-2-Hexenal	17	Green, banana, aldehydic, fatty, cheesy
4	(E)-2-Hexenol	1	Resin, flower, green
5	Decanal	2	Soap, orange peel, tallow
6	Linalool	7.4	Sweet, floral, citrus
7	Undecanal	5	Sweet
8	(E)-2-Decenal	0.35	Waxy, fatty, earthy, coriander-like, green, mushroom, aldehydic
9	Dodecanal	2	Floral
10	(E)-2-Undecenal	5	Fresh, fruity, citrus, orange peel
11	(E)-2-Dodecenal	1.4	Green, fat, sweet
12	(E)-2-Tridecenal	0.8	Flower, sweet, must

*The compounds and their corresponding ID can be identified on the example chromatogram in Supplementary Figure 1.*

**FIGURE 3:**
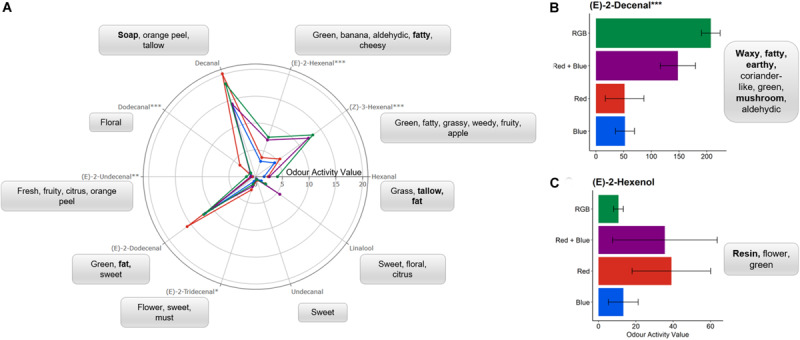
**(A)** An aroma wheel depicting the odour activity values (OAVs) of 10 of the 12 aromatic compounds identified in *C. sativum* grown under blue (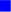), red (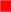), RB (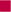), or RGB (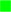) spectrums (see Figure 1 and section “Materials and Methods” for details of wavelengths). One-way ANOVAs were performed for each compound and the corresponding *P*-value with significance between growth spectra treatments indicated by asterisks (***P < 0.0001, **P < 0.001, *P < 0.05, *n* = 4). Each compound is associated with the generally accepted descriptors; with unpleasant scents highlighted in bold. For clarity, the mean OAVs of **(B)** (E)-2-decenal and **(C)** (E)-2-hexenol are plotted separately with standard deviation. Bar charts of the mean OAV and standard deviation for each compound are shown in Supplementary Figure S3.

Of the compounds identified using the GC-O, six were found to significantly differ in relative concentration between plants grown under specific LED spectrums (Figure 3).

### The Impact of Specific Spectrum on the Aroma and Physiology of *C. sativum*

Light-emitting diode lamps emitting four different spectrums were used to determine the impact of specific wavelengths on the concentration of aromatic compounds in fresh, above-ground *C. sativum* material grown in controlled environment settings (Figure 1 and Supplementary Figure S2). The intensity of the lamps was kept constant during the daytime phase at 150 μmol m^–2^ s^–1^ PPFD. In general, the greater the number of wavelengths included in the treatment, the higher the odour activity of the compound; however, there were notable exceptions (Figure 3 and Supplementary Figure S3).

Plants grown under red LEDs demonstrated significant increases in (E)-2-tridecenal and dodecanal when compared to the other LED treatments. For dodecanal, this activity was significantly higher for the plants grown under red light when compared to all other LED treatments (*P* < 0.003) while for (E)-2-tridecenal this increase in activity was only significantly higher than those found in the material grown under blue LEDs (*P* = 0.013). Dodecanal and (E)-2-tridecenal are responsible for the “Soapy, waxy, aldehydic, citrus, orange rind with floral nuances” and “sweet” scents from fresh coriander (Table 1). These long chain aldehydes have the smallest odour thresholds (Table 1) and therefore, at higher concentrations, would have a greater contribution to the overall scent perceived by the sniffer and potentially favor of the fresh material. The compounds with the lowest odour threshold and most associated with the scent of fresh coriander, (E)-2-decenal and (Z)-3-hexenal, were significantly (*P* < 0.01) higher in the RGB and RB treatments when compared with the monochromatic spectrums. With the addition of green wavelengths, the relative concentration of (E)-2-decenal was significantly (*P* = 0.03) higher in the RGB grown plants when compared to the RB treatment.

**FIGURE 4:**
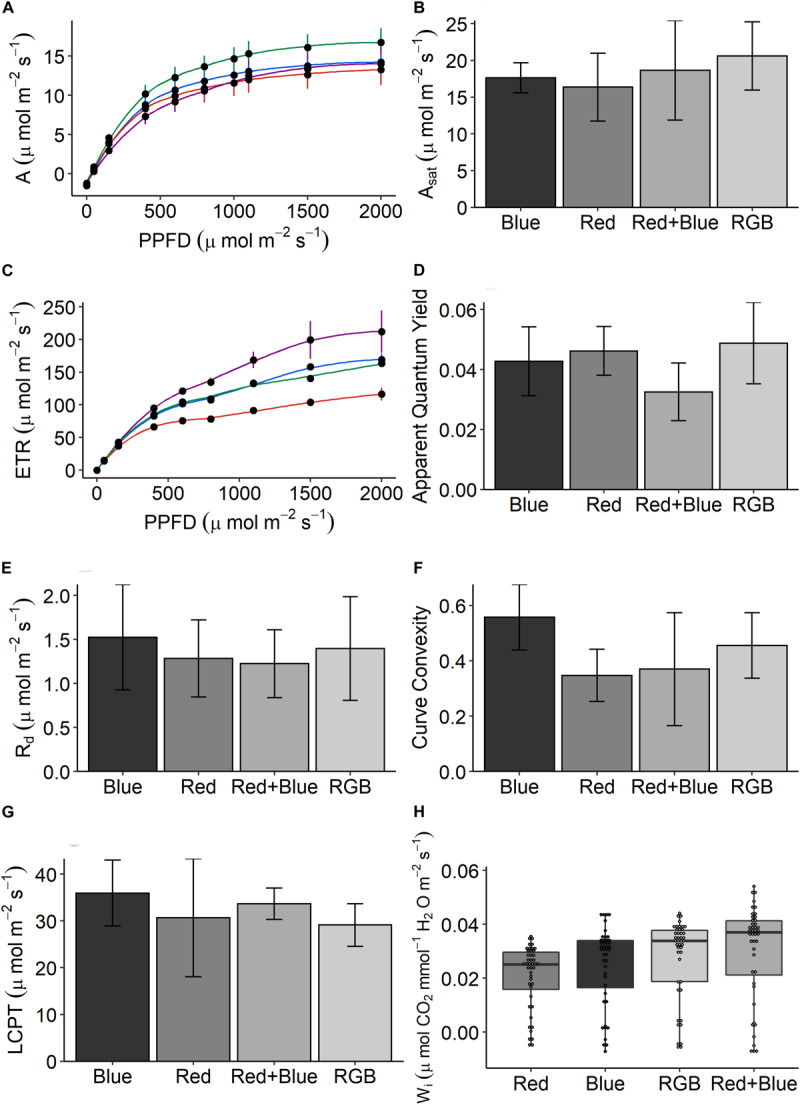
The response of **(A)** net photosynthetic CO_2_ assimilation (*A*) in response to increasing photosynthetic photon flux density (PPFD) for plants grown under the four LED spectrums. From these response curves, **(B)** light-saturated rates of A (*A*_sat_), **(C)** the response of electron transport (ETR), **(D)** apparent quantum yield (mol CO_2_/mol quanta), **(E)** the rate of respiration (R_d_), **(F)** curve convexity, and **(G)** light compensation point (LCPT) can be estimated. Finally, **(H)** intrinsic water-use efficiency (W_i_)—the ratio of CO_2_ assimilated to water lost via stomatal conductance—is calculated for every replicate under each PPFD intensity. Data are the means with standard deviations (*n* = 4).

Overall, the data suggest that plants grown under RB and RGB had increased photosynthetic capacity and W_i_ but there are some inconsistencies. Plants grown under the RGB wavelengths achieved the highest rates of CO_2_ assimilation (*A*—Figures 4A,B) and apparent quantum yield (Figure 4D) under saturating PPFD conditions, while the plants grown under the single wavelengths showed the lowest. This indicates that RGB could drive higher potential plant growth rate in comparison with other wavelengths. Interestingly, plants grown under RB wavelengths consistently achieved the highest rates of electron transport (ETR—Figure 4C). No significant differences for any of photosynthetic parameters were observed between the plants grown under the four different LED treatments (Figures 4A–G, *P* > 0.05). The respiration (Figure 4E), measure of curve convexity (Figure 4F), and LCPT (Figure 4G) were highest in the leaves grown under blue LEDs.

Intrinsic W_i_—the ratio of CO_2_ assimilated to water lost via stomatal conductance—was calculated under each PPFD intensity for each treatment (Figure 4H). On average, plants grown under the dichromatic RB spectrum achieved the highest W_i_ due to significantly lower stomatal conductance (*P* < 0.0001), this was especially clear at the low PPFD intensities (<1000 μmol m^–2^ s^–1^ PPFD—Supplementary Figure S4). Plants grown under the red spectrum demonstrated the lowest W_i_, significantly (*P* < 0.0001) lower than plants grown under the blue, RB, and RGB spectrums.

**FIGURE 5:**
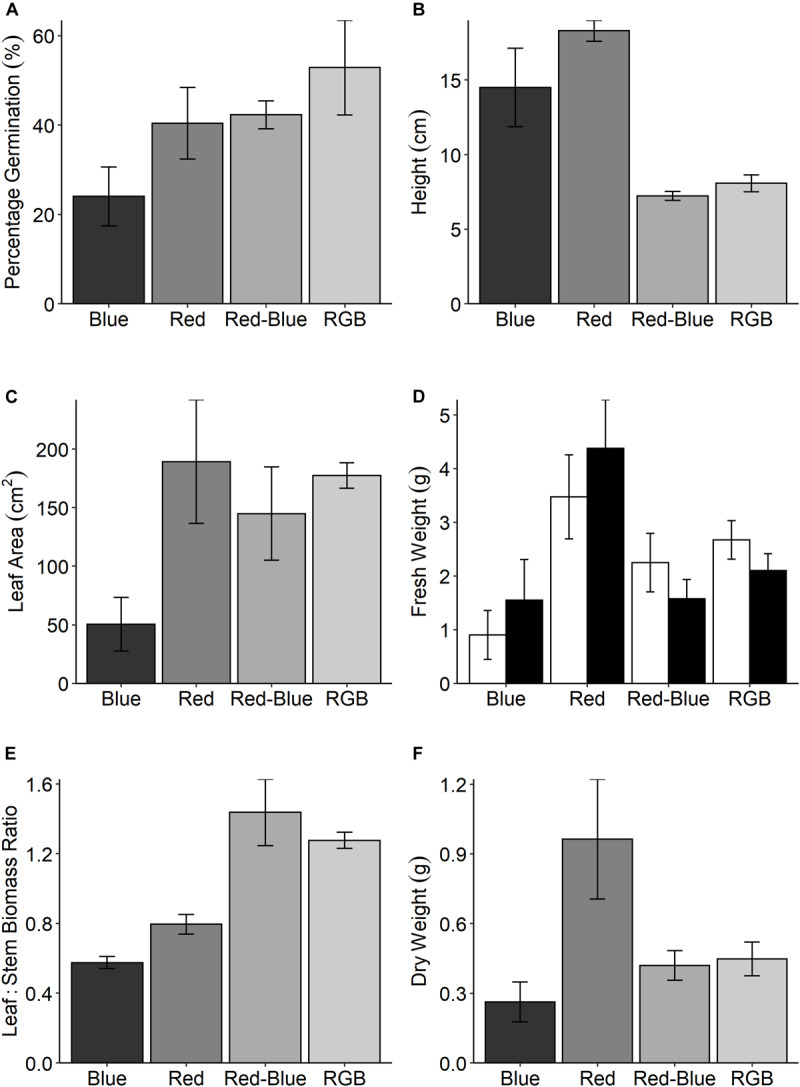
**(A)** The percentage germination for *C. sativum* grown under four spectral mixes; red, blue, RB, and RGB (see Figure 1 and section “Materials and Methods” for specific wavelengths). At 21 days, the height of these plants were measured **(B)** and all above ground material was assessed for **(C)** leaf area and **(D)** fresh weight for both the leaves (□) and stems (■). The leaf:stem biomass **(E)** was calculated from these values. Finally, the **(F)** dry weight was determined. Data are the means with standard deviation (*n* = 4).

In the production of commercial coriander the grower selects plants with a desirable stem:leaf ratio; while short plants are appealing to the grower for transportation, leafy plants are selected by the consumer for inclusion in recipes. To assess these morphological characteristics, we measured plant features following growth under the four specific LED treatments (Figure 1). Although all plants received the same PPFD, significant differences in above ground biomass were determined between the treatments for all harvest traits (Figure 5). Despite low germination rates (Figure 5A), plants grown under the red spectrum were the tallest (Figure 5B), achieved the highest leaf area (Figure 5C) and total biomass (stem and leaf—Figure 5D) and dry weight (Figure 5F). Interestingly, these plants demonstrated the lowest rates of CO_2_ assimilation (Figure 4A). Plants grown under the RB and RGB spectrums had the greatest leaf to stem ratios (Figure 4E, 1.44 ± 0.19 and 1.28 ± 0.05, respectively) while plants grown under the singular red and blue spectrums had significantly lower leaf:stem ratios (0.58 ± 0.04 and 0.8 ± 0.06, respectively). We conclude that the shorter stature of the RB and RGB treatments tend toward an optimal morphology due to the smaller stem length under these spectra.

### Coriander Grown in Commercial Glasshouse Settings

**FIGURE 6:**
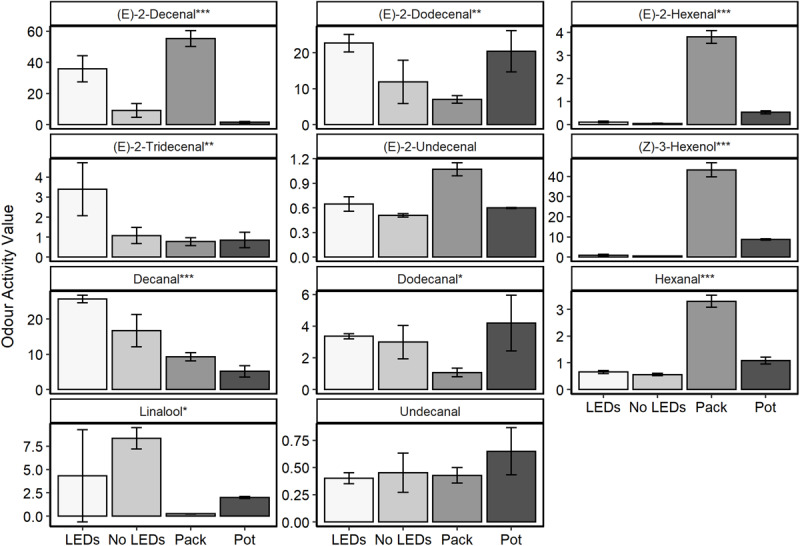
The odour activity values (concentration in ppb/odour threshold) of nine compounds of interest harvested from fresh *C. sativum* material (*n* = 3) commercially grown under LED supplemental lighting with near equal contributions of red, green, and blue wavelengths (35.8% red: 26.4% green: 37.8% blue, respectively—LEDs) and no supplemental lighting (No LEDs) or from tissue sampled from supermarket leaves, either a sealed packet (Pack) or growing in a pot (Pot). One-way ANOVAs were performed for each compound and the corresponding *P*-value with significance between treatments indicated by asterisks on the panel titles (***P < 0.0001, **P < 0.001, *P < 0.05).

Aromatic compounds previously identified as characteristic of the coriander aroma (Table 1) were relatively quantified for plant material grown in a commercial glasshouse with no supplemental lighting and for plants grown under LED lamps supplementing approximately equal thirds red, green, and blue wavelengths (35.8% red: 26.4% green: 37.8% blue, respectively) in a commercial glasshouse (Figure 1). These results were also compared with two supermarket products; coriander sold in a pot and excised coriander leaves sold in sealed packets (Figure 6 and Supplementary Figure S5).

Significant differences were noted between the treatments for 10 of the 11 aromatic compounds identified (*P* < 0.03). The plants grown under LED lighting demonstrated the highest concentrations of the 11–13 carbon chain aldehydes [(E)-2-dodecanal and (E)-2-tridecenal] and the highest (*P* < 0.0001) concentration of decanal—the compound that characterizes both favorable nuances such as citrus and florals but is also responsible for the soapy undertones of coriander aroma (Table 1). Decenal was found to be lowest in material harvested from the purchased, potted coriander, significantly lower than in the material grown with or without supplemental LED lighting (*P* < 0.003).

The packets of excised coriander purchased from a supermarket showed the highest concentrations of six-carbon aldehydes and alcohols: (E)-2-hexanal, hexanal, and (Z)-3-hexenol. These compounds are associated with the “green” and “grassy” nuances of coriander aroma (Table 1). Interestingly, for six out of 11 of the aromatics, concentrations were higher in the excised, packet coriander than in the potted supermarket coriander.

In general, the plants grown in a commercial glasshouse with no supplemental light were found to have lower concentrations of the aromatic compounds of interest when compared to the material grown under the LEDs. The one exception to this observation was linalool, which was found to be 1.9-fold higher in these plants and over 33-fold higher than measured in the packaged supermarket leaves.

## Discussion

While past studies have focused on differences in the aromatic composition of *C. sativum*, for example, comparing seeds and leaves (Shahwar et al., 2012), wild and domestic varieties (Eyres et al., 2005) or post-harvest treatment (Buthelezi et al., 2016), very few have stated growing conditions of the samples and how these environmental conditions may impact on aroma. Here, we present a study on the combined impact of spectra on the aroma and physiology of commercial *C. sativum.*

Our findings have determined that the majority of *C. sativum* high impact aromatics reported in the literature (Table 1) were also observed for United Kingdom-based, commercially grown coriander as determined using the NIF panel and GC-O chromatogram (Figure 2 and Supplementary Figure 1). Using these compounds as a gauge, significant differences in concentration (Figure 3) were noted when coriander was grown under distinct LED spectra (Figure 1), emitting the same PPFD, in controlled environment growth rooms. This indicates that the aroma of these plants was modified by wavelength and not total PPFD. This observation has also been noted in the literature for the herbs basil (*Ocimum basilicum*), dill (*Anethum graveolens*), and parsley (*Petroselinum crispum*) (Litvin-Zabal, 2019; Pennisi et al., 2019). For *C. sativum*, while adding greater numbers of narrow band wavelengths generally increased the concentration of most aromatics, significant differences were also observed for plants grown under single wavebands (Figure 3). This suggests that it is possible to manipulate the concentration of particular compounds through the addition or removal of particular wavelengths. For example, to increase the intensity of aromatics most associated with *C. sativum* (e.g., E-2-decenal) for a more desirable stronger scent or decrease compounds that are less desirable (e.g., decenal, dodecanal), which are associated with waxy, soapy nuances (Table 1).

### Growth, Morphology, and Spectrum

Manipulating spectra also generates marked changes in key physiological (Figure 4) and morphological (Figure 5) characteristics. While not significant in this dataset, growing crops under specific spectrums are known to manipulate photosynthesis and associated processes. All three wavebands (red, blue, and green) are capable of driving photosynthesis with the same quantum efficiency (McCree, 1971; Hogewoning et al., 2012); however, differences in chlorophyll content and leaf/canopy structure (Murchie and Horton, 1997; Terashima et al., 2010) dictate the proportions of each waveband that will reach the chloroplast, depending on position in the leaf and the canopy. Although no anatomical measurements were taken for this paper, this might explain the waveband specific variation in photosynthetic parameters observed in Figure 4. It is also possible that at higher growth PPFD, the differences between the photosynthetic responses observed under each spectrum would induce irradiance acclimation responses (Murchie and Horton, 1997) and hence become much greater, as a supplement of 150 μmol m^–2^ s^–1^ PPFD is low compared to the daily light received in a glasshouse, depending on latitude and season.

Plants respond to signals derived from light quality and quantity, mediated by an array of photoreceptors such as phytochrome and cryptochrome (Smith et al., 2017). The complex morphological responses observed here, and the associated photosynthetic properties will at least partly be a result of such signaling processes. It is hard to de-convolute these with the current data set and this was not the intention of the study but here we explore possible biological processes at work. Although the plants grown under the red-only spectrum demonstrated some of the lowest rates of photosynthesis per unit leaf area (Schepens et al., 2004), they also demonstrated the highest accumulation of fresh and dry biomass (Figures 5D,F). A likely explanation for this is that these plants allocated greater resources to above ground biomass accumulation and leaf area, while CO_2_ assimilation was reduced due to lower amounts of photosynthetic components (such as the enzyme Ribulose bis-phoshate carboxylase/oxygenase or Rubisco) per unit leaf area (Bugbee and Salisbury, 1988; Kim et al., 2004). In fact, a higher leaf area with lower specific leaf weight (thinner leaves) and increased stem elongation is a common strategy to enhance light capture in low light growing environments (Evans and Poorter, 2001) and explains leaf and stem morphology within the current growth PPFD. A lack of investment in photosynthetic assimilation per unit leaf area would limit plants grown under red wavelengths if the total PPFD exceeded 350 μmol m^–2^ s^–1^ while those plants grown under RB or RGB mixes would better utilize higher light—potentially accumulating greater biomass more rapidly (Figure 4A). Changes in leaf area and stem elongation under low irradiance have been linked to activity of the photoreceptor phytochrome which is determined by the ratio of red to far red light. We suggest such a response is at work here (Franklin and Whitelam, 2005). In contrast, blue light effects are mediated by the photoreceptor cryptochrome (Smith et al., 2017) which, among other effects, is known to regulate leaf specific leaf weight (thickness). Thus addition of blue light can lead to thick leaves with higher amounts of photosynthetic components and a higher photosynthetic capacity but this effect is hard to discern in the current data set. The large differences seen in Figure 5 between red, blue, and RB are likely to be a result if interactions between these different photoreceptor-mediated processes. The particularly high rate of photosynthesis in the RGB plants is hard to explain but may be a result of enhanced Rubisco content per unit leaf area as a result of photoacclimation to red, blue, and green. Green light is known to interact with blue light responses (Smith et al., 2017). This study only includes single measurements at one developmental stage and does not include lifetime measurements of CO_2_ assimilation or dark respiration, which may also explain some of the disparity in biomass acquisition between the treatments.

While green light is known to reverse blue-light stimulated stomatal opening (Frechilla et al., 2000; Smith et al., 2017), the plants grown under RB had the highest mean intrinsic W_i_ (Figure 4H). It is well known that red and blue wavelengths modulate stomatal behavior (Lawson, 2009), with a small amount of blue on a red background initiating rapid stomatal opening (Shimazaki et al., 2007). For coriander grown under a 50:50 red:blue ratio, this effect seems negated by the concurrent lower stomatal conductance and higher rates of CO_2_ assimilation demonstrating the highest W_i_ between the spectrum treatments. In addition to the regulation of W_i_, green light has been implicated for improving CO_2_ uptake in lower levels of the canopy (Smith et al., 2017)—coriander develops a highly dense canopy prior to harvest and the addition of green wavelengths to supplemental lighting may increase growth rates and maintain better canopy structure in packed commercial glasshouse growing conditions.

### Aroma and Growth Spectrum

It is less clear how such mechanisms could influence the flavor and aroma properties, but it seems likely that they are involved too: for example, phytochrome is implicated at all major developmental transitions in plants in addition to environmental signal processing (Schepens et al., 2004). Further, red to far red ratios are known to regulate volatile organic compound emissions such as ethylene, in response to plant to plant competition (Kegge and Pierik, 2010).

**FIGURE 7:**
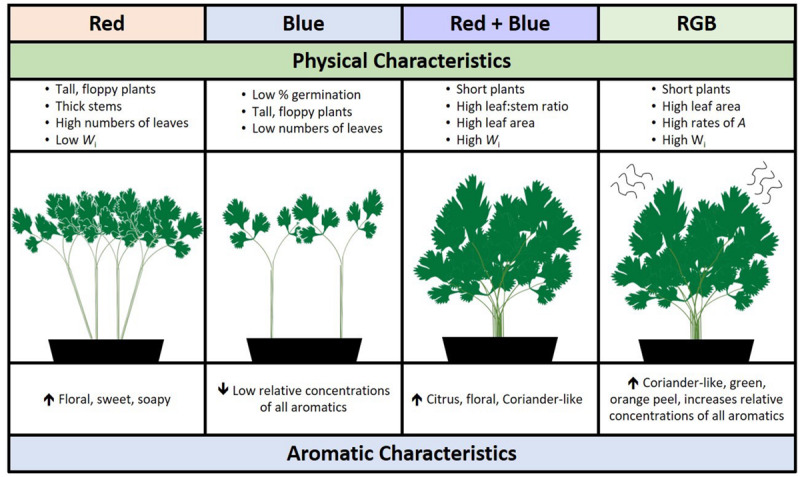
A schematic summarizing the overall impact of specific wavebands on the morphological and aromatic characteristics of *C. sativum* grown under red, blue, RB, and RGB LEDs (see Figure 1 and section “Materials and Methods” for details of wavelengths).

In addition to strong aroma, the fresh coriander ideotype for commercial production would have high seed fecundity (i.e., high germination rates), produce large numbers of leaves to stems, and be easy to transport to supermarkets. Interestingly, plants grown under red wavelengths acquired the greatest biomass in the same period of time but were also significantly taller suggesting a leggy, shade-avoidance phenotype (Fraser et al., 2016). Plants grown under red-only wavelengths also produce higher concentrations of unpleasant aromatics decenal and E-(2)-tridecenal (soapy, sweet, and musty aromatic nuances). While greater numbers of wavelengths had a positive impact on germination and leaf area, the RB spectrum improved the leaf:stem ratio while shortening the plants, potentially making them easier to pack or harvest. From these results, we conclude that there is a fine balance between manipulating aroma and maintaining the preferred coriander ideotype; using specific LED supplements may yield the best aroma but not a visually appealing plant (Figure 7). This may allow growers to target niche products such as curry pastes, micro-greens, or custom vertical farming, a growing global market (Vertical farming research predicts industry growth, 2019). Such options are dependent on the ability of growers to invest in lighting infrastructure for industrial scale production, assuming a viable business model.

As well as manipulating the aroma of low odour threshold aromatics in controlled growth room conditions, glasshouse-based supplemental LED lighting was also found to manipulate the odour profile of coriander (Figure 6). With the exception of linalool and undecanal, using LEDs to supplement the light of glasshouse-grown coriander increased the concentration of all the aromatics measured when compared to non-supplemented plants. Comparing LED-supplemented material to non-Supplemented Material uncovered significant differences in the quantity of high impact aromatics including decanal and (E)-2-decenal which would potentially make the supplemented coriander smell (and taste) more strongly of coriander. However, increases in decenal, although contributing stronger citrusy undertones to the material, could also discourage some consumers due to increases in a soapy aroma.

The data presented here also suggest that coriander which has been cut and packed for sale has a different aromatic composition to the coriander sold in a pot. Although this could be attributed to the samples originating from different companies or production batches, increases in long-chain aldehydes and alcohols have been linked to the stress response of plants (Holopainen and Gershenzon, 2010). Increases in the concentration of long-chain aldehydes in the cut material could also reflect the material beginning to dry after excision, therefore concentrating the aromatics before our GC-MS analysis (hence higher relative concentrations) and promoting the intensification of the aromatics after purchase in the supermarket for the consumer. This also suggests potential to manipulate aroma profile of the coriander in post-harvest processing by irradiating the excised material under specific spectrum or by selling excised material grown under specific spectrum.

The highest concentrations of linalool, a volatile monoterpene (Schwab et al., 2008), were determined for the plants grown in a glasshouse without supplemental LED lighting. Linalool is a natural herbivore repellent indirectly defending against herbivory and acting as an attractant to predators of herbivores (Degenhardt et al., 2003). High concentrations of this compound in the leaves of *Arabidopsis thaliana* and potato have been shown to repel aphids and mites through manipulation of the terpenoid biosynthesis pathway (Kappers et al., 2005; Aharoni et al., 2006). The presence of linalool in these particular samples suggests defense against biotic stresses (Holopainen and Gershenzon, 2010) in the glasshouse environment. With less linalool detected in the plants grown under supplemental LEDs, or in the packed or potted supermarket coriander, these data suggest that these plants may be more susceptible to biotic attack within glasshouse or growth room setups. In addition to plant defenses, the terpenoid pathways have also been manipulated in tomato plants to engineer more aromatic and flavorful fruits (Lewinsohn et al., 2001). Metabolic engineering of volatile terpenoids can have negative implications for plant growth and fitness (Aharoni et al., 2003, 2006), leading to lowered potential oil yields in some species (Mahmoud and Croteau, 2001). This suggests that the decrease of linalool in LED grown plants will also lead to a changed perception of *C. sativum* aroma for the consumer, which may or may not be negated by the increase of lower threshold, similar smelling compounds.

To our knowledge, this is the first publication investigating the combined impact of spectra on the physiology, growth and aroma of *C. sativum* plants. These data highlight the fine balance between actively manipulating the growth and physiology to produce a marketable plant while maintaining or strengthening the aromatics in this popular herb.

## Conclusion

The work presented here and, in the literature, suggests that modification of aroma in response to growth spectrum is an adaptive response that is linked to numerous diverse biological roles. The observations that aromatic complexity derives from spectral complexity may appear surprising, but we must recall that the existence of desirable secondary plant compounds is related to defense, competition, and fitness. When we move from a monochromatic source, which the plant is unlikely to experience in nature, to more complex dichromatic and trichromatic spectra we may progressively be activating the signaling pathways that give rise to these measurable economic traits. In this paper, we hypothesize that we have de-convoluted these pathways through the manipulation of growing spectra. The practical application of our observations is that flavor and aroma can be co-manipulated with the form (morphology) of the plant, enabling a grower to produce a combination according to market demand and this principle has a biological basis.

## Data Availability Statement

The raw data supporting the conclusions of this article will be made available by the authors, without undue reservation, to any qualified researcher.

## Author Contributions

EM, IF, JC, and LM conceived this project. DM designed and managed the manufacture of the LED lamps. M-TL performed and analyzed the gas chromatography mass spectrometry and olfactometry data. LM and HS-H grew the plants, performed all the phenotypic experiments in the laboratory, and sampled. UM managed the field experiment at Langmeads Farm and sampled the plants. LM wrote the manuscript. All authors reviewed the final manuscript.

## Conflict of Interest

The authors declare that the research was conducted in the absence of any commercial or financial relationships that could be construed as a potential conflict of interest.

## Footnotes

1 http://www.r-project.org/

## Supplementary Material

FIGURE S1 A representative chromatograms demonstrating the relative abundance of aromatic compounds found in above-ground *C. sativum* material purchased from a commercial retailers (*n* = 3). The numbers above each peak indicate the presence of the aromatic compounds (Table 1 for details), indicated by the NIF test and literature as being a main constituent of the aroma of fresh coriander leaves.

FIGURE S2 Representative chromatograms of above-ground *C. sativum* material grown in controlled environment chambers under 50 μmol m^–2^ s^–1^ photosynthetic photon flux density (PPFD) of either (a) red, (b) blue, (c) RB, or (d) RGB LEDs (see Figure 1 and section “Materials and Methods” for details of wavelengths) (*n* = 4).

FIGURE S3 The odour activity values (concentration in ppb/odour threshold) of 12 compounds of interest as determined from fresh *C. sativum* grown under blue, red, RB, or RGB spectrums. Data are the means (*n* = 4) with standard deviation. One-way ANOVAs were performed for each compound and the corresponding *P*-value with significance between growth spectra treatments indicated by asterisks (****P* < 0.0001, ***P* < 0.001, **P* < 0.05).

FIGURE S4 The relationship between CO_2_ assimilation (*A*) and stomatal conductance (*g*_s_) for plants grown under four separate spectrums; red (), blue (), RB (), and RGB () (see Figure 1 and section “Materials and Methods” for details of wavelengths) in response to changes in PPFD (*n* = 4).

FIGURE S5 Representative chromatograms of *C. sativum* material sampled from four sources; **(A)** commercially sold *C. sativum* from a pot, **(B)** commercially sold *C. sativum* leaves, excised and sold in a packet, **(C)** *C. sativum* grown in a commercial glasshouse under supplemental LEDs, and **(D)** *C. sativum* grown in a commercial glasshouse with no supplemental LEDs.

## Abbreviations

*A*: rate of photosynthetic CO_2_ assimilation, μ mol m^–2^ s^–1^
*A*_sat_: light-saturated rate of photosynthetic CO_2_ assimilation, μ mol m^–2^ s^–1^
GC-MS: gas chromatography mass spectrometry
GC-O: gas chromatography olfactometry
LED: light-emitting diode
NIF: nasal impact frequency
OAV: odour activity value
PFD: photon flux density, μ mol m^–2^ s^–1^
PPFD: photosynthetic photon flux density (400–700 nm only) μ mol m^–2^ s^–1^
RB: red + blue treatment
RGB: red + green + blue treatment
W_i_: water-use efficiency, μ mol CO_2_ mmol^–1^ H_2_O m^–2^ s^–1^.

## References

[B1] Acree T. E. Heinrich A. (2004). *Flavornet and Human Odor Space.* Available at: http://flavornet.org/index.html (Accessed August 1, 2019).

[B2] Acree T. E. Teranishi R. (1993). *Flavor Science: Sensible Principles and Techniques.* Washington, DC: American Chemical Society.

[B3] Aharoni A. Giri A. P. Deuerlein S. Griepink F. de Kogel W.-J. Verstappen F. W. (2003). Terpenoid metabolism in wild-type and transgenic *Arabidopsis* plants. *Plant Cell* 15 2866–2884. 10.1105/tpc.016253 14630967 PMC282818

[B4] Aharoni A. Jongsma M. A. Kim T.-Y. Ri M.-B. Giri A. P. Verstappen F. W. (2006). Metabolic engineering of terpenoid biosynthesis in plants. *Phytochem. Rev.* 5 49–58.

[B5] Alrifai O. Hao X. Marcone M. F. Tsao R. (2019). Current review of the modulatory effects of LED lights on photosynthesis of secondary metabolites and future perspectives of microgreen vegetables. *J. Agric. Food Chem.* 67 6075–6090. 10.1021/acs.jafc.9b00819 31021630

[B6] Briggs W. R. Christie J. M. (2002). Phototropins 1 and 2: versatile plant blue-light receptors. *Trends Plant Sci.* 7 204–210. 10.1016/s1360-1385(02)02245-8 11992825

[B7] Brown J. (2020). *Haz-Map Database*. Available online at: https://haz-map.com/About (accessed October 2018).

[B8] Bugbee B. G. Salisbury F. B. (1988). Exploring the limits of crop productivity: I. Photosynthetic efficiency of wheat in high irradiance environments. *Plant Physiol.* 88 869–878. 10.1104/pp.88.3.869 11537442 PMC1055676

[B9] Buthelezi M. N. D. Soundy P. Jifon J. Sivakumar D. (2016). Spectral quality of photo-selective nets improves phytochemicals and aroma volatiles in coriander leaves (*Coriandrum sativum* L.) after postharvest storage. *J. Photochem. Photobiol. B Biol.* 161 328–334. 10.1016/j.jphotobiol.2016.05.032 27295414

[B10] Colquhoun T. A. Schwieterman M. L. Gilbert J. L. Jaworski E. A. Langer K. M. Jones C. R. (2013). Light modulation of volatile organic compounds from petunia flowers and select fruits. *Postharvest Biol. Technol.* 86 37–44. 10.1016/j.postharvbio.2013.06.013

[B11] Crepy M. A. Casal J. J. (2015). Photoreceptor-mediated kin recognition in plants. *New Phytol.* 205 329–338. 10.1111/nph.13040 25264216

[B12] Curutchet A. Dellacassa E. Ringuelet J. A. Chaves A. R. Viña S. Z. (2014). Nutritional and sensory quality during refrigerated storage of fresh-cut mints (*Mentha× piperita* and *M. spicata*). *Food Chem.* 143 231–238. 10.1016/j.foodchem.2013.07.117 24054235

[B13] Davis P. A. Burns C. (2016). Photobiology in protected horticulture. *Food Energy Security* 5 223–238. 10.1002/fes3.97 15115293

[B14] Degenhardt J. Gershenzon J. Baldwin I. T. Kessler A. (2003). Attracting friends to feast on foes: engineering terpene emission to make crop plants more attractive to herbivore enemies. *Curr. Opin. Biotechnol* 14 169–176. 10.1016/s0958-1669(03)00025-9 12732318

[B15] Demarsy E. Goldschmidt-Clermont M. Ulm R. (2018). Coping with ‘dark sides of the sun’through photoreceptor signaling. *Trends Plant Sci.* 23 260–271. 10.1016/j.tplants.2017.11.007 29233601

[B16] Diederichsen A. (1996). *Coriander: Coriandrum Sativum L*, Vol. 3 Rome: Bioversity International.

[B17] Dudareva N. Negre F. Nagegowda D. A. Orlova I. (2006). Plant volatiles: recent advances and future perspectives. *Crit. Rev. Plant Sci.* 25 417–440. 10.1080/07352680600899973

[B18] Evans J. Poorter H. (2001). Photosynthetic acclimation of plants to growth irradiance: the relative importance of specific leaf area and nitrogen partitioning in maximizing carbon gain. *Plant Cell Environ.* 24 755–767. 10.1046/j.1365-3040.2001.00724.x

[B19] Eyres G. Dufour J. P. Hallifax G. Sotheeswaran S. Marriott P. J. (2005). Identification of character-impact odorants in coriander and wild coriander leaves using gas chromatography-olfactometry (GCO) and comprehensive two-dimensional gas chromatography–time-of-flight mass spectrometry (GC× GC–TOFMS). *J. Separat. Sci.* 28 1061–1074. 10.1002/jssc.200500012 16013833

[B20] Fan X. Sokorai K. J. (2002). Changes in volatile compounds of γ-irradiated fresh cilantro leaves during cold storage. *J. Agric. Food Chem.* 50 7622–7626. 10.1021/jf020584j 12475280

[B21] Franklin K. A. Whitelam G. C. (2005). Phytochromes and shade-avoidance responses in plants. *Ann. Bot.* 96 169–175. 10.1093/aob/mci165 15894550 PMC4246865

[B22] Fraser D. P. Hayes S. Franklin K. A. (2016). Photoreceptor crosstalk in shade avoidance. *Curr. Opin. Plant Biol.* 33 1–7. 10.1016/j.pbi.2016.03.008 27060719

[B23] Fraser K. Singleton I. Borland A. (2015). *Investigating the Cause and Potential Treatment of Coriander Yield Decline.* Pittsburgh, PA: School of Natural and Environmental Sciences.

[B24] Frechilla S. Talbott L. D. Bogomolni R. A. Zeiger E. (2000). Reversal of blue light-stimulated stomatal opening by green light. *Plant Cell Physiol.* 41 171. 10.1093/pcp/41.2.171 10795311

[B25] Genty B. Briantais J. M. Baker N. R. (1989). The relationship between the quantum yield of photosynthetic electron transport and quenching of chlorophyll fluorescence. *Biochim. Biophys. Acta Gen. Subj.* 990 87–92. 10.1016/s0304-4165(89)80016-9

[B26] Heberling J. M. Fridley J. D. (2013). Resource-use strategies of native and invasive plants in Eastern North American forests. New Phytol. 200 523-533. 10.1111/nph.12388 23815090

[B27] Hogewoning S. W. Wientjes E. Douwstra P. Trouwborst G. Van Ieperen W. Croce R. (2012). Photosynthetic quantum yield dynamics: from photosystems to leaves. *Plant Cell* 24 1921–1935. 10.1105/tpc.112.097972 22623496 PMC3442578

[B28] Holopainen J. K. Gershenzon J. (2010). Multiple stress factors and the emission of plant VOCs. *Trends Plant Sci.* 15 176–184. 10.1016/j.tplants.2010.01.006 20144557

[B29] Kappers I. F. Aharoni A. Van Herpen T. W. Luckerhoff L. L. Dicke M. Bouwmeester H. J. (2005). Genetic engineering of terpenoid metabolism attracts bodyguards to *Arabidopsis*. *Science* 309 2070–2072. 10.1126/science.1116232 16179482

[B30] Kegge W. Pierik R. (2010). Biogenic volatile organic compounds and plant competition. *Trends Plant Sci.* 15 126–132. 10.1016/j.tplants.2009.11.007 20036599

[B31] Kim H.-H. Goins G. D. Wheeler R. M. Sager J. C. (2004). Stomatal conductance of lettuce grown under or exposed to different light qualities. *Ann. Bot.* 94 691–697. 10.1093/aob/mch192 15347557 PMC4242213

[B32] Kong S.-G. Okajima K. (2016). Diverse photoreceptors and light responses in plants. *J. Plant Res.* 129 111–114. 10.1007/s10265-016-0792-5 26860414

[B33] Larner V. S. Franklin K. A. Whitelam G. C. (2008). 5 Photoreceptors and light signalling pathways in plants. *Annu. Plant Rev. Endog. Plant Rhythms* 21:107 10.1002/9781119312994.apr0210

[B34] Lawson T. (2009). Guard cell photosynthesis and stomatal function. *New Phytol.* 181 13–34. 10.1111/j.1469-8137.2008.02685.x 19076715

[B35] Lee M. K. Arasu M. V. Park S. Byeon D. H. Chung S.-O. Park S. U. (2016). LED lights enhance metabolites and antioxidants in chinese cabbage and kale. *Braz. Arch. Biol. Technol.* 59:e1615 0546.

[B36] Leffingwell and Associates, (1985). Available at: https://www.leffingwell.com/flavbase.htm (Accessed September 24, 2019).

[B37] Lewinsohn E. Schalechet F. Wilkinson J. Matsui K. Tadmor Y. Nam K.-H. (2001). Enhanced levels of the aroma and flavor compound S-linalool by metabolic engineering of the terpenoid pathway in tomato fruits. *Plant Physiol.* 127 1256–1265. 10.1104/pp.010293 11706204 PMC129293

[B38] Litvin-Zabal A. G. (2019). *Quantifying the Effects of Light Quantity and Quality on Culinary Herb Physiology.* Ann Arbor, MI: ProQuest Dissertations Publishing.

[B39] Macleod A. J. Islam R. (1976). Volatile flavour components of coriander leaf. *J. Sci. Food Agric.* 27 721–725. 10.1002/jsfa.2740270803 16358762

[B40] Mahmoud S. S. Croteau R. B. (2001). Metabolic engineering of essential oil yield and composition in mint by altering expression of deoxyxylulose phosphate reductoisomerase and menthofuran synthase. *Proc. Natl. Acad. Sci. U.S.A.* 98 8915–8920. 10.1073/pnas.141237298 11427737 PMC37535

[B41] Marshall B. Biscoe P. (1980). A model for C3 leaves describing the dependence of net photosynthesis on irradiance. *J. Exp. Bot.* 31 29–39. 10.1093/jxb/31.1.29

[B42] Massa G. D. Kim H.-H. Wheeler R. M. Mitchell C. A. (2008). Plant productivity in response to LED lighting. *HortScience* 43 1951–1956. 10.21273/hortsci.43.7.1951

[B43] McCree K. J. (1971). The action spectrum, absorptance and quantum yield of photosynthesis in crop plants. *Agric. Meteorol.* 9 191–216. 10.1016/0002-1571(71)90022-7

[B44] Murchie E. Horton P. (1997). Acclimation of photosynthesis to irradiance and spectral quality in British plant species: chlorophyll content, photosynthetic capacity and habitat preference. *Plant Cell Environ.* 20 438–448. 10.1046/j.1365-3040.1997.d01-95.x

[B45] Pennisi G. Blasioli S. Cellini A. Maia L. Crepaldi A. Braschi I. (2019). Unravelling the role of red: blue LED lights on resource use efficiency and nutritional properties of indoor grown sweet basil. *Front. Plant Sci.* 10:305 10.3389/fpls.2019.00305 PMC6424884 30918510

[B46] RStudio Team, (2015). *RStudio: Integrated Development for R.* Boston, MA: RStudio, Inc.

[B47] Sahib N. G. Anwar F. Gilani A. H. Hamid A. A. Saari N. Alkharfy K. M. (2013). Coriander (*Coriandrum sativum* L.): a potential source of high-value components for functional foods and nutraceuticals-a review. *Phytother. Res.* 27 1439–1456. 10.1002/ptr.4897 23281145

[B48] Samuolienë G. Brazaitytë A. Viršilë A. Jankauskienë J. Sakalauskienë S. Duchovskis P. (2016). Red light-dose or wavelength-dependent photoresponse of antioxidants in herb microgreens. *PLoS One* 11:e0163405. 10.1371/journal.pone.0163405 27677090 PMC5038936

[B49] Samuolienë G. Sirtautas R. Brazaitytë A. Duchovskis P. (2012). LED lighting and seasonality effects antioxidant properties of baby leaf lettuce. *Food Chem.* 134 1494–1499. 10.1016/j.foodchem.2012.03.061 25005972

[B50] Schepens I. Duek P. Fankhauser C. (2004). Phytochrome-mediated light signalling in *Arabidopsis*. *Curr. Opin. Plant Biol.* 7 564–569. 10.1016/j.pbi.2004.07.004 15337099

[B51] Schwab W. Davidovich-Rikanati R. Lewinsohn E. (2008). Biosynthesis of plant-derived flavor compounds. *Plant J.* 54 712–732. 10.1111/j.1365-313x.2008.03446.x 18476874

[B52] Shahwar M. K. El-Ghorab A. H. Anjum F. M. Butt M. S. Hussain S. Nadeem M. (2012). Characterization of coriander (*Coriandrum sativum* L.) seeds and leaves: volatile and non volatile extracts. *Int. J. Food Propert.* 15 736–747. 10.1080/10942912.2010.500068

[B53] Shimazaki K. I. Doi M. Assmann S. M. Kinoshita T. (2007). Light regulation of stomatal movement. *Annu. Rev. Plant Biol.* 58 219–247. 10.1146/annurev.arplant.57.032905.105434 17209798

[B54] Smith H. L. (2017). *Optimisation of Light Spectral Quality to Improve Plant Growth and Development.* Nottingham: University of Nottingham.

[B55] Smith H. L. McAusland L. Murchie E. H. (2017). Don’t ignore the green light: exploring diverse roles in plant processes. *J. Exp. Bot.* 68 2099–2110. 10.1093/jxb/erx098 28575474

[B56] Takahashi S. Badger M. R. (2011). Photoprotection in plants: a new light on photosystem II damage. *Trends Plant Sci.* 16 53–60. 10.1016/j.tplants.2010.10.001 21050798

[B57] Terashima I. Hanba Y. T. Tholen D. Niinmets U. (2010). Leaf functional anatomy in relation to photosynthesis. *Plant Physiol.* 155 108–116. 10.1104/pp.110.165472 21075960 PMC3075775

[B58] The Good Scents Company [tgsc] (2018). Available at: http://www.thegoodscentscompany.com/index.html (Accessed September 19, 2019).

[B59] Van Gemert L. (2003). *Odour Thresholds. Compilations of Odour Threshold Values in Air, Water and Other Media Oliemans.* Utrecht: Punter & Partners BV.

[B60] Vertical farming research predicts industry growth, (2019). *Government Europa.* Available at: https://www.governmenteuropa.eu/vertical-farming-market-research-report/92426/ (accessed August 28, 2019).

[B61] Zhang M. Whitman C. M. Runkle E. S. (2019). Manipulating growth, color, and taste attributes of fresh cut lettuce by greenhouse supplemental lighting. *Sci. Hortic.* 252 274–282. 10.1016/j.scienta.2019.03.051

