# Supplementary figures and images for: Growth Spectrum Complexity Dictates Aromatic Intensity in Coriander (Coriandrum sativum L.)
Source: Front Plant Sci. 2020 May 15;11:462. doi: 10.3389/fpls.2020.00462 (PMC7242725; doi:10.3389/fpls.2020.00462)

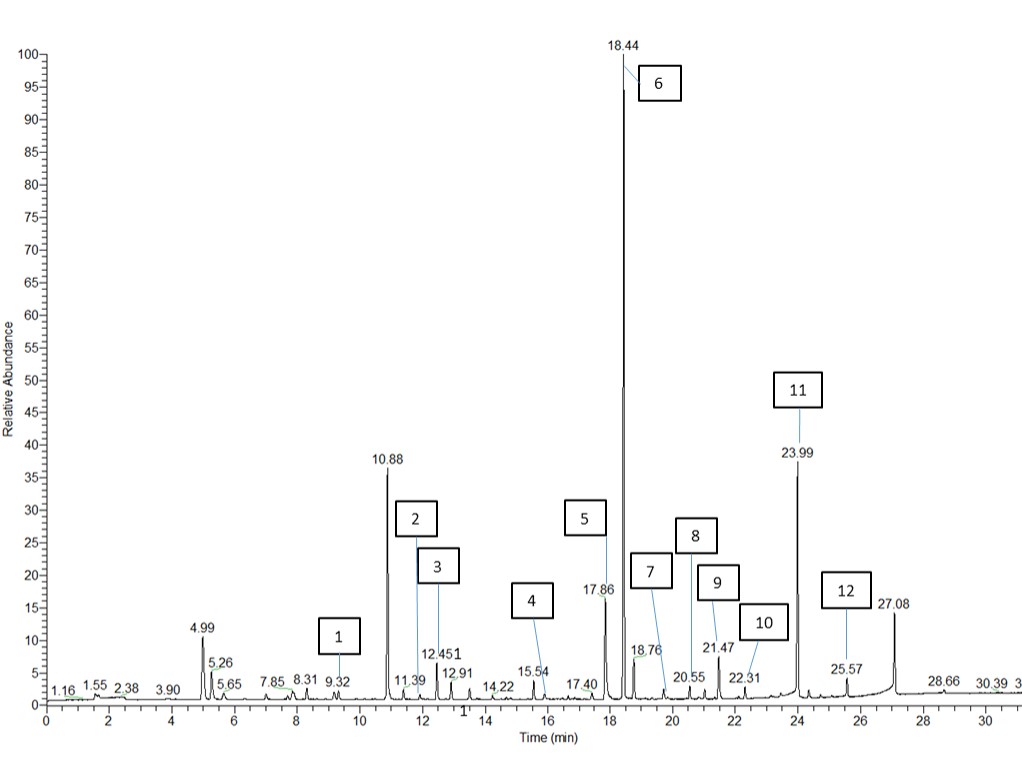

Supplement: FIGURE S1 — A representative chromatograms demonstrating the relative abundance of aromatic compounds found in above-ground C. sativum material purchased from a commercial retailers (n = 3). The numbers above each peak indicate the presence of the aromatic compounds (Table 1 for details), indicated by the NIF test and literature as being a main constituent of the aroma of fresh coriander leaves. [file Image_1.JPEG]

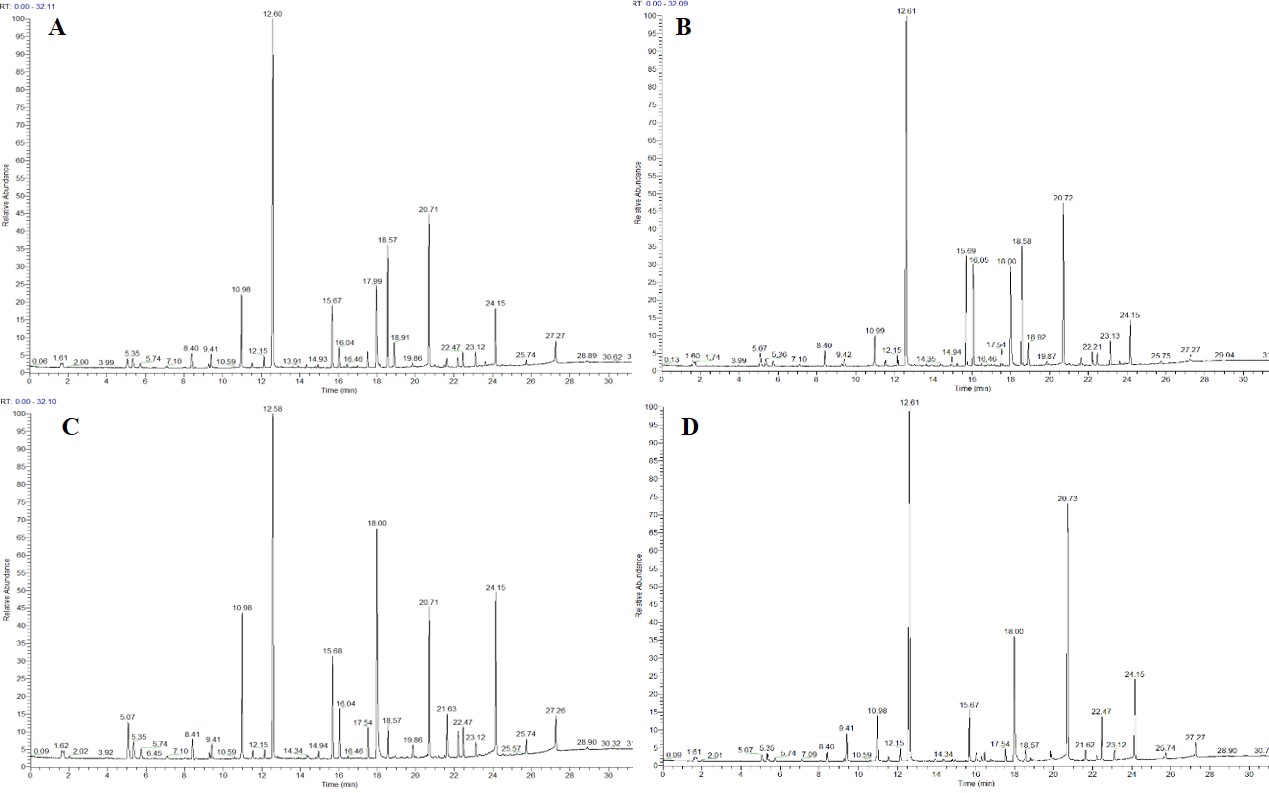

Supplement: FIGURE S2 — Representative chromatograms of above-ground C. sativum material grown in controlled environment chambers under 50 μmol m–2 s–1 photosynthetic photon flux density (PPFD) of either (a) red, (b) blue, (c) RB, or (d) RGB LEDs (see Figure 1 and section “Materials and Methods” for details of wavelengths) (n = 4). [file Image_2.JPEG]

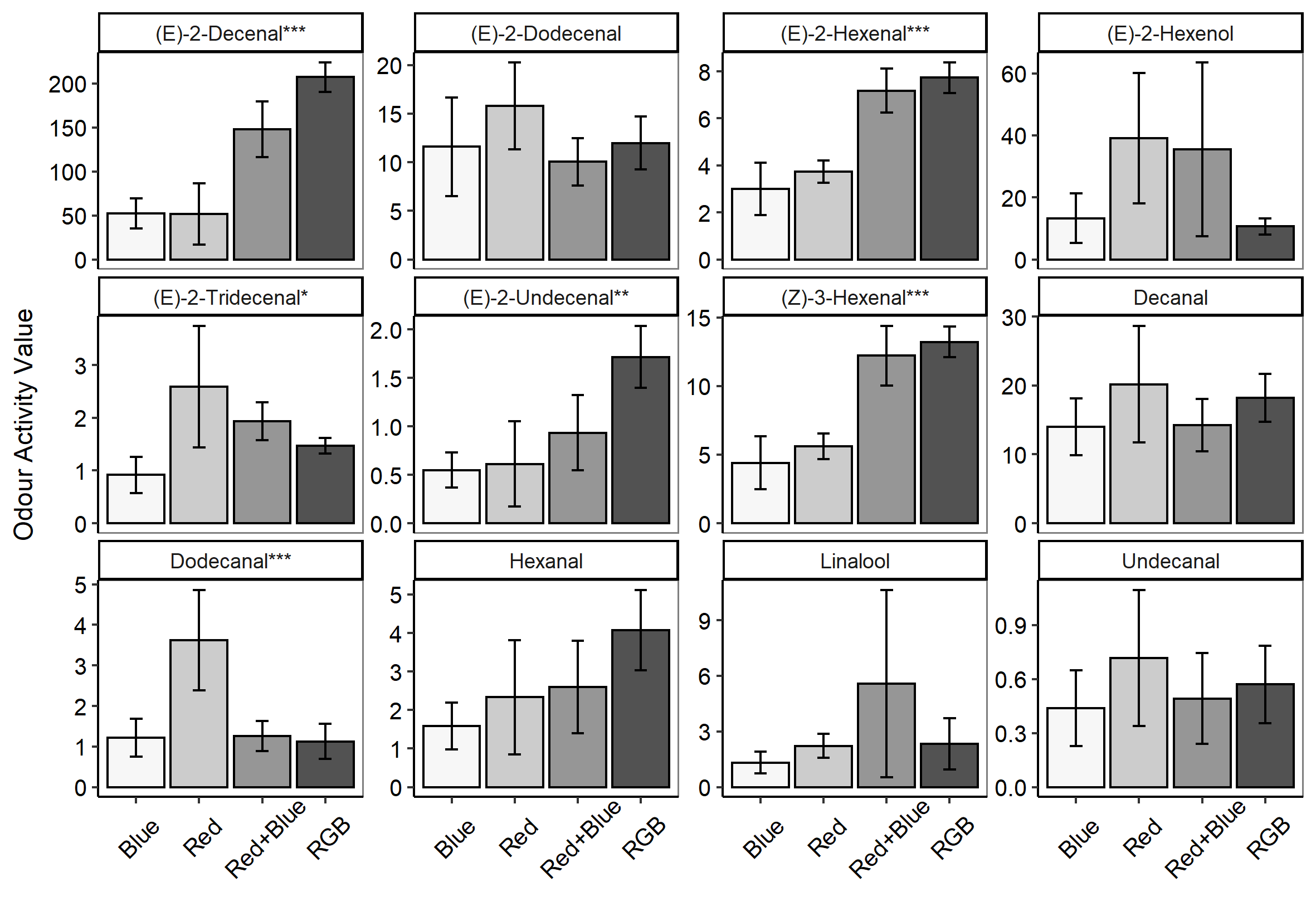

Supplement: FIGURE S3 — The odour activity values (concentration in ppb/odour threshold) of 12 compounds of interest as determined from fresh C. sativum grown under blue, red, RB, or RGB spectrums. Data are the means (n = 4) with standard deviation. One-way ANOVAs were performed for each compound and the corresponding P-value with significance between growth spectra treatments indicated by asterisks (***P < 0.0001, **P < 0.001, *P < 0.05). [file Image_3.TIFF]

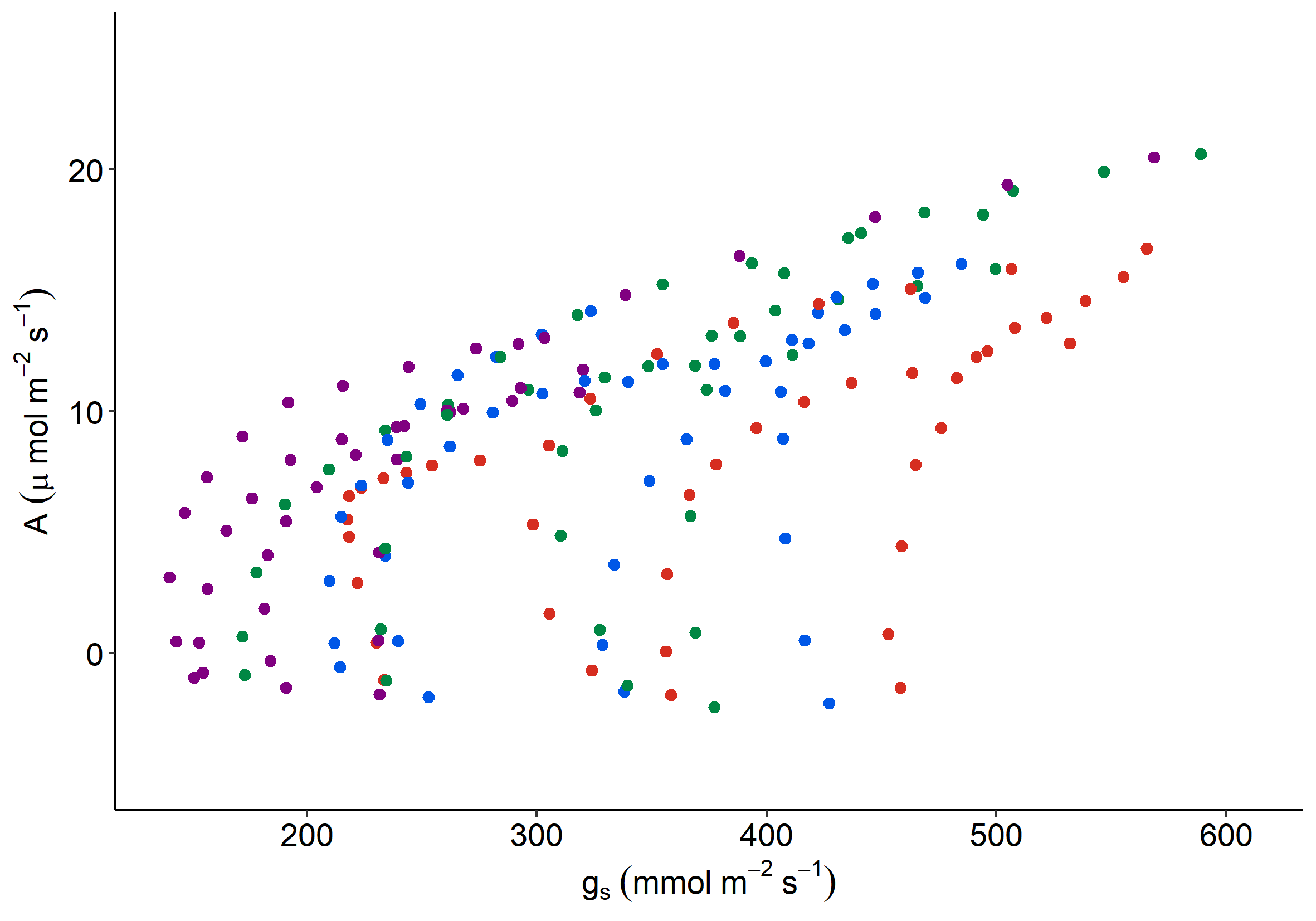

Supplement: FIGURE S4 — The relationship between CO2 assimilation (A) and stomatal conductance (gs) for plants grown under four separate spectrums; red (), blue (), RB (), and RGB () (see Figure 1 and section “Materials and Methods” for details of wavelengths) in response to changes in PPFD (n = 4). [file Image_4.TIFF]

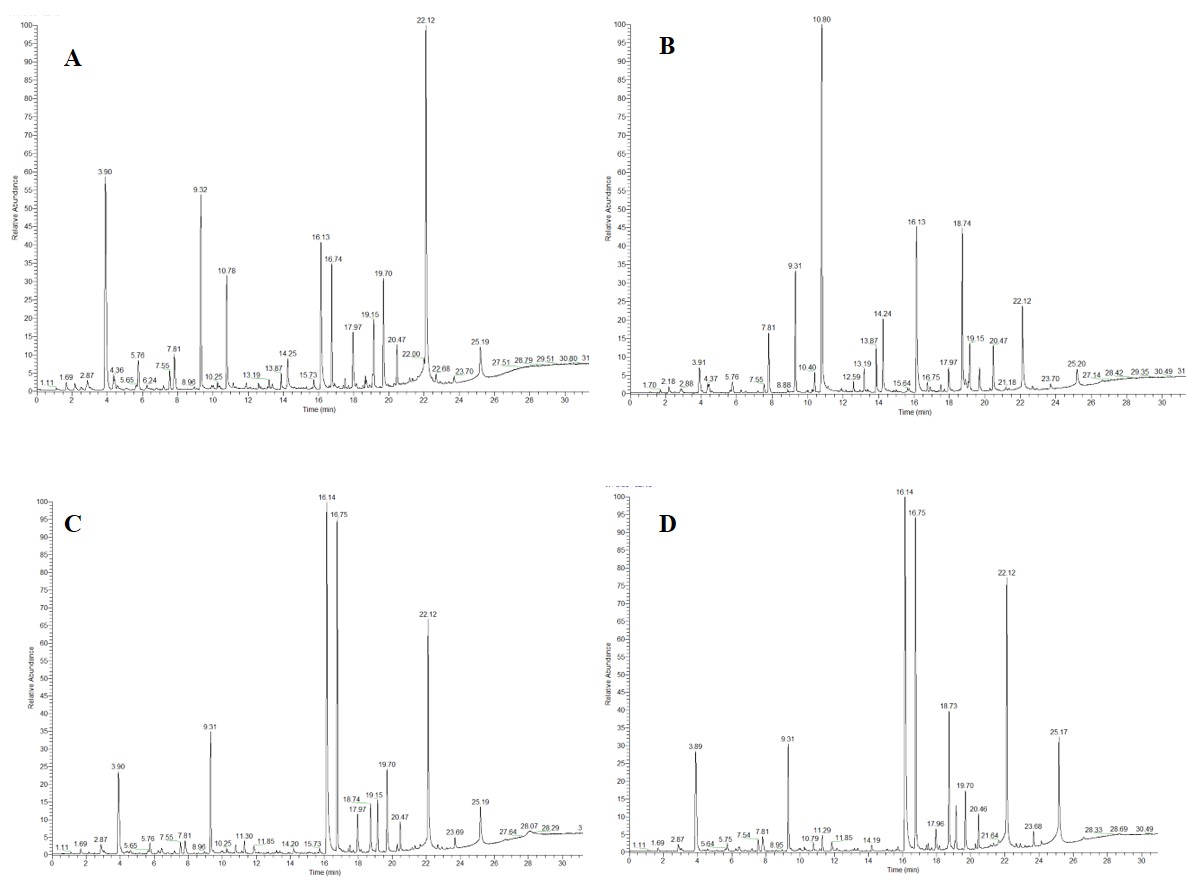

Supplement: FIGURE S5 — Representative chromatograms of C. sativum material sampled from four sources; (A) commercially sold C. sativum from a pot, (B) commercially sold C. sativum leaves, excised and sold in a packet, (C) C. sativum grown in a commercial glasshouse under supplemental LEDs, and (D) C. sativum grown in a commercial glasshouse with no supplemental LEDs. [file Image_5.JPEG]
